# Ruptured popliteal artery aneurysm in a patient with a clinical diagnosis of Marfan syndrome

**DOI:** 10.1590/1677-5449.200017

**Published:** 2020-10-16

**Authors:** Gabriel Paiva Duarte, Jorge Ribeiro da Cunha

**Affiliations:** 1 Universidade Federal Fluminense – UFF, Faculdade de Medicina, Niterói, RJ, Brasil.; 2 Hospital Estadual Alberto Torres – HEAT, São Gonçalo, RJ, Brasil.

**Keywords:** popliteal artery, ruptured aneurysm, Marfan syndrome

## Abstract

The popliteal artery is the main site of occurrence of peripheral aneurysms. Acute presentations constitute a potential threat to limb viability and to life, especially in the event of rupture. Rupture is a rare event, but one that demands an immediate intervention decision to achieve a satisfactory treatment outcome. The gold standard treatment is conventional surgery, effecting repair by interposition of a great saphenous vein graft. Studies conducted in recent decades have found associations between Marfan Syndrome and peripheral aneurysms. This report presents a case of a ruptured left popliteal artery aneurysm successfully treated in an 82-year-old patient clinically diagnosed with previously unknown Marfan syndrome.

## INTRODUCTION

Popliteal artery aneurysm (PAA) is the most common type of peripheral aneurysm^1^^,^^2^ and constitutes a threat to the limb involved and, in more severe cases, to the life of the patient. PAAs may be asymptomatic^3^ or may manifest acutely as medical emergencies. Thromboembolic phenomena are the most common causes of emergencies and ruptures are rarer.^2^^-^^5^

Etiology is primarily linked to atherosclerosis,^1^ but can also be related to inflammatory states, infectious conditions, and connective tissue diseases.^6^ Prevalence is higher among men and the elderly,^2^ and the primary risk factors are arterial hypertension and smoking.^4^^,^^5^ Development of PAAs in patients with Marfan Syndrome (MFS) is rare, and few cases have been reported.^7^^-^^9^ Gaertner et al.^10^ identified one patient with a PAA among 15 MFS patients who underwent vascular examination using ultrasound.

## CASE DESCRIPTION

**Figure 1:**
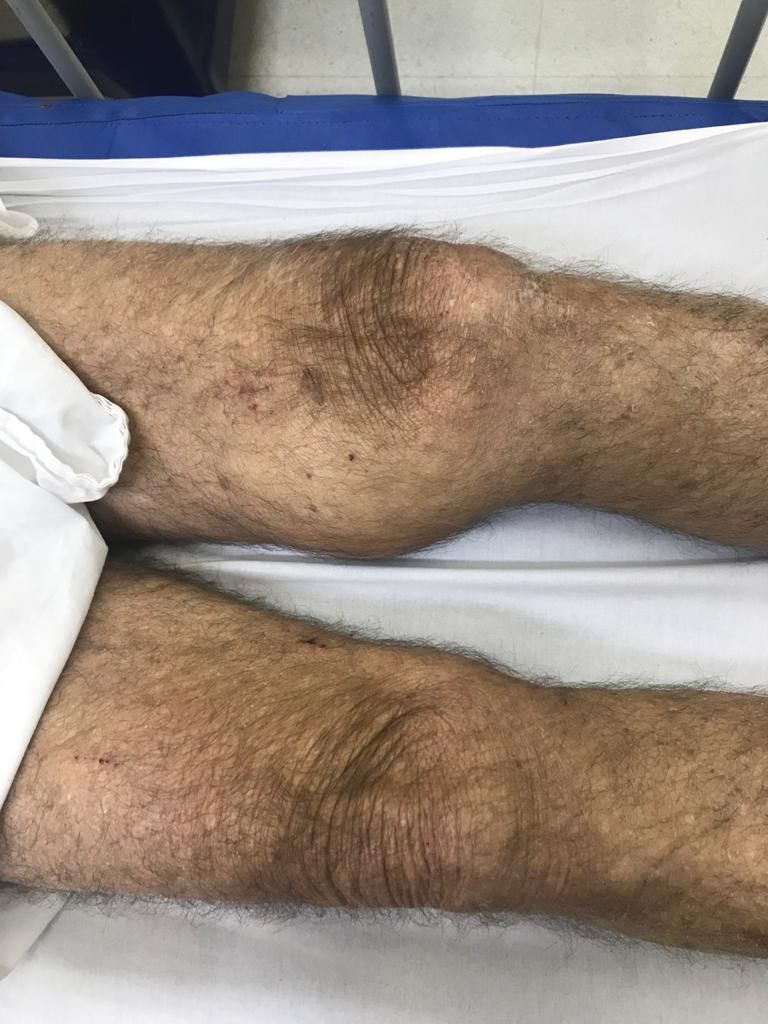
Patient before operation.

An 82-year-old male patient presented at the emergency department complaining of pain in his left leg. He reported painful swelling in both popliteal fossae that had emerged 2 years previously and increased in size thereafter. Around two days previously, the swelling on the left had enlarged significantly and become more painful. Physical examination revealed a pulsatile tumor in the left popliteal region (Figure 1).

**Figure 2:**
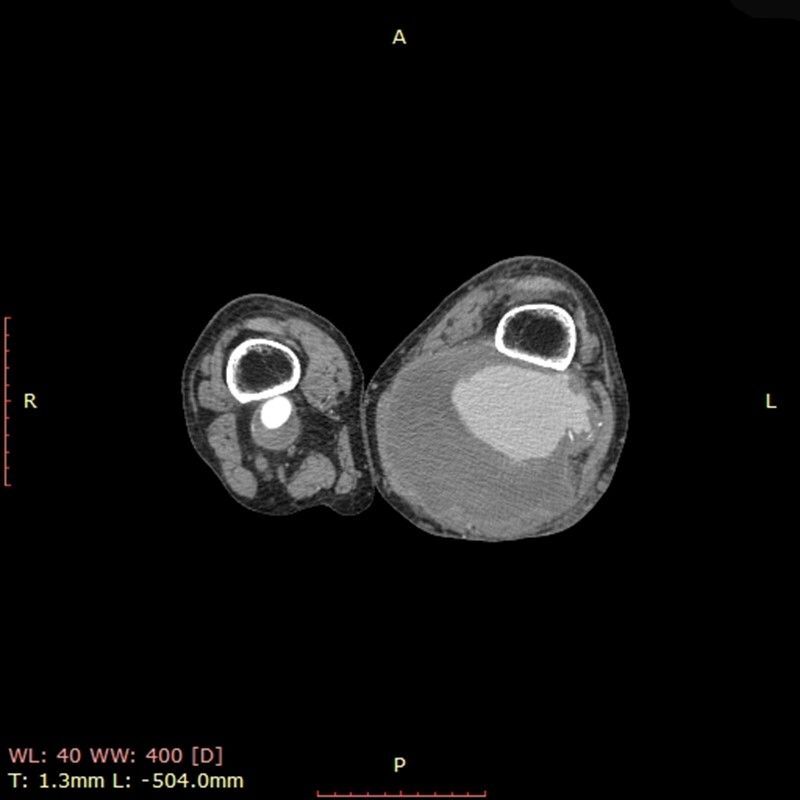
Lower limb computed tomography angiography with contrast showing left and right popliteal artery aneurysms. The left aneurysm has ruptured.

The patient underwent computed tomography (CT) with contrast, showing an enlarged left popliteal artery (LPA), with thrombus and contrast leakage delimited by a sac (Figure 2), findings characteristic of a ruptured left PAA measuring 14.4 centimeters at its greatest diameter. As soon as laboratory test results had been checked and the patient had been informed of the situation, he was transferred to the operating room for immediate repair of the ruptured aneurysm.

**Figure 3:**
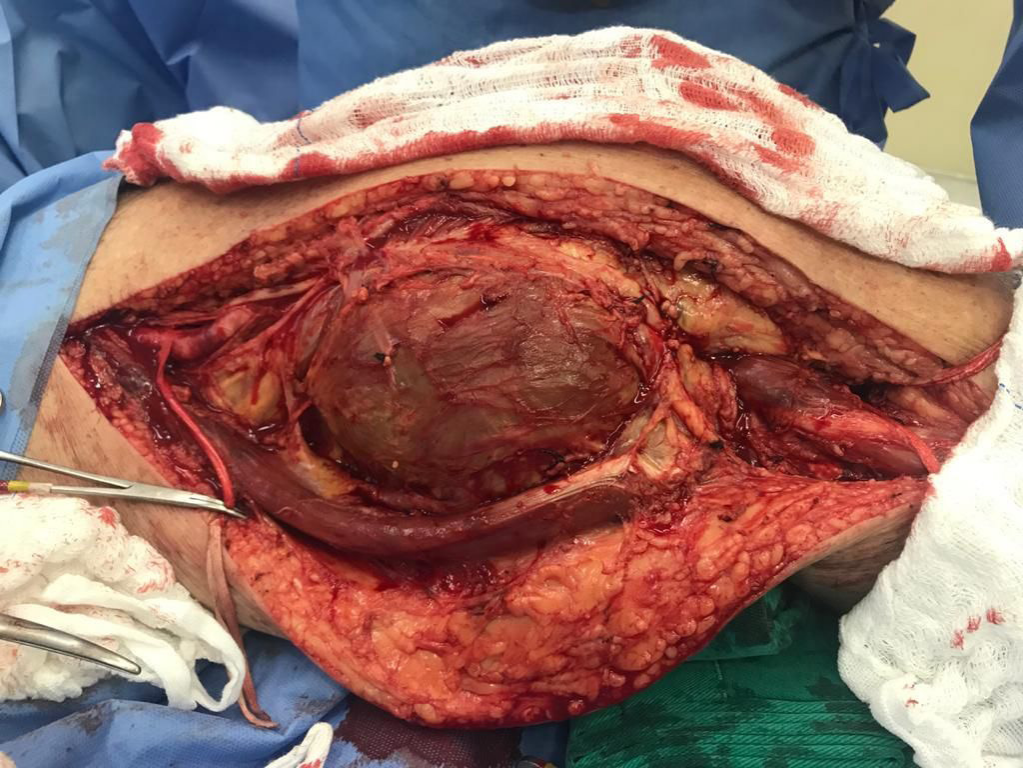
Left popliteal artery aneurysm sac dissected with control of proximal and distal blood flow.

**Figure 4:**
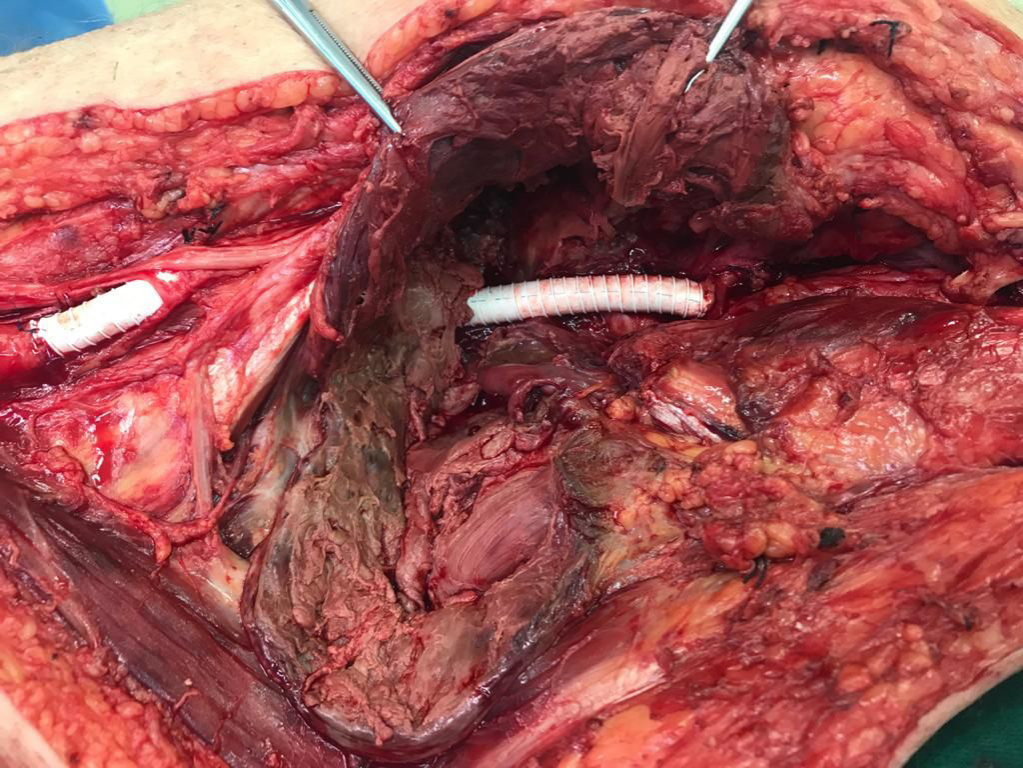
End-to-end anastomosis of the left popliteal artery with interposition of a polytetrafluoroethylene (PTFE) graft.

The approach chosen was via medial access in the thigh and leg, with the patient in dorsal decubitus under general anesthesia. The aneurysm sac was identified and then dissected until the proximal and distal portions of the LPA were identified. Both were clamped to control proximal and distal arterial flow (Figure 3). The aneurysm sac was incised and its thrombotic contents removed. A ringed polytetrafluoroethylene (PTFE) graft was interposed between the proximal and distal segments of the LPA (Figure 4). The patient had previously undergone saphenectomy, precluding use of his great saphenous vein (GSV) for grafting.

**Figure 5:**
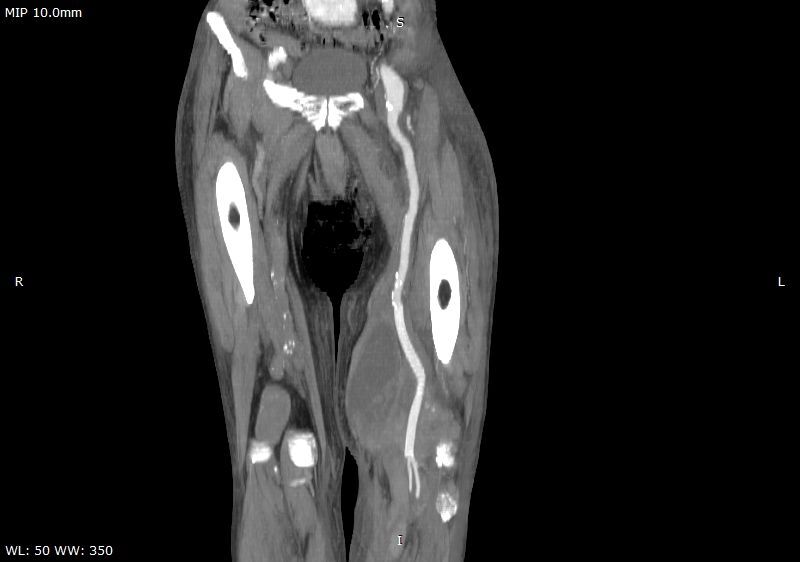
Postoperative coronal image showing patent graft.

The patient was transferred to the intensive care unit (ICU). He had persistent leukocytosis during his stay in the ICU and we considered the possibility of a graft infection. On the 7th day in the ICU, he underwent another CT of the lower extremities with contrast. This examination showed that the graft, the distal LPA, and its terminal branches, the anterior tibial artery, the posterior tibial artery, and the fibular artery, were all patent (Figure 5). These images ruled out the hypothesis of graft infection. The patient’s leukocytosis improved after antibiotic therapy to treat pneumonia and he was discharged from hospital 14 days after surgery.

During the initial physical examination, we identified physical characteristics of MFS - arachnodactyly and enophthalmos. One week after surgery, another physical examination was conducted, following the revised Ghent Nosology diagnostic criteria for MFS.^11^ The patient had dilation of the aortic root, with a diameter of 48 millimeters and an aortic root z score of 4.19. The following findings were also present: wrist and thumb sign, valgus hindfoot deformity, thoracic asymmetry, thoracolumbar scoliosis, and protrusio acetabuli. These findings, in conjunction with a z score > 2 fulfill the criteria for a clinical diagnosis of MFS.

## DISCUSSION

The popliteal artery is the most common site of peripheral aneurysms (70 to 85%).^1^^,^^3^ Among people who have a PAA, there is also a high prevalence of aneurysms at other sites in the body, such as the contralateral popliteal artery (40 to 68.9%) and the abdominal aorta (30 to 60%).^3^^-^^5^^,^^12^^,^^13^ Development of PAA is related to atherosclerotic disease and occurs in individuals with well-defined characteristics: male patients, from the 7th decade of life onwards. The most closely related risk factors are arterial hypertension and smoking, and less closely linked factors include traumas, inflammatory states, syphilis infection, and connective tissue diseases, such as Marfan and Ehlers-Danlos syndromes.^4^^,^^6^^,^^12^^,^^13^

Clinically, PAAs can present gradually or acutely. Progressive presentations present with intermittent claudication and with compressive symptoms, or may even be asymptomatic. The most common acute presentations or complications are linked to restriction of arterial flow, caused by thrombosis of the artery or distal embolization. Symptoms include pallor, absent distal pulses, and sudden loss of strength in the limb.^1^ Aneurysmal rupture is a rare complication in PAAs (0.5 to 7%).^1^ Acute PAA presentations should be treated as medical emergencies. Prompt resolution is necessary because limb viability is threatened and because of the potential threat to the patient’s life. The diagnosis is confirmed using imaging exams capable of showing in detail the location and morphology of the injury. CT with contrast, duplex ultrasonography, magnetic resonance imaging (MRI), and angiography are the exams of choice.^3^^,^^14^

Prompt reestablishment of perfusion of the limb is the basis of treatment for any of the acute complications of a PAA. There are two groups of treatment: conventional surgery (CS) and endovascular repair (ER).^3^^,^^14^ CS consists of anastomosis of the two segments of the vessel, proximal and distal to the injury, or by interposition of an autologous or prosthetic graft. The GSV is the autologous graft material most often used, while the prosthetic graft material most often used is PTFE. In our case, we decided to use a ringed PTFE graft because the distal anastomosis was beyond the knee joint line and a ringed graft is less likely to kink than a non-ringed graft. ER techniques are based on stenting the site of injury or embolization of the aneurysm.

The gold standard treatment is CS and this should be chosen for the majority of patients.^3^^,^^4^ ER is preferred for patients with elevated surgical risk, because it is a less invasive method.^6^^,^^14^ The most common postoperative complications are related to hospitalization (respiratory tract infections, acute renal failure, deep venous thrombosis), followed by graft thrombosis, hematoma involving the surgical wound, and problems with healing.^3^ Use of ER enables patients to spend less time in hospital and is associated with lower rates of surgical wound complications. In counterpoint, CS is associated with lower risk of thrombosis of venous or prosthetic grafts and better primary patency of the anastomosis over the long term.^15^

A meta-analysis by Leake et al.^15^ found a 3-year primary patency rate of 79.4% for CS, whereas the equivalent patency rate for ER was 68.2%. Aulivolla et al.^5^ reported 5-year primary patency rates of 85% for CS and 44.4% for ER. Restricting the analysis to the CS treatment group, Huang et al.^4^ showed that interposition of the GSV was superior to PTFE grafts in terms of 5-year primary patency. Patency using the GSV was 85%, whereas with PTFE grafts it was 50%.

It appears that PAAs are part of a group of aneurysmal disorders that also affect arteries in other parts of the body. After surgical repair, 49% of patients will develop another aneurysm within 10 years.^2^ These patients should undergo vascular system investigation as routine.^2^

## MARFAN SYNDROME

MFS is a connective tissue disease caused by a mutation in the FBN1 gene causing deformities involving the bones and the pulmonary, cardiovascular, and ocular systems.^16^ The FBN1 gene mutation in MFS causes changes to the fibrillin protein, which is one of the components of the extracellular matrix of arterial connective tissues. The vessel wall dilatations seen in people with the syndrome are attributed to consequences of this mutation.^16^

One of the conditions associated with MFS is dilatation of the ascending aorta, although there are few reports of aneurysms in other arteries. We found reports of aneurysmal dilatations in other segments of the aorta, in visceral branches, pulmonary arteries, and peripheral arteries in patient with MFS, but few reports relating the syndrome to PAAs.^7^^-^^9^^,^^17^^-^^19^

Yetman et al.^17^ conducted a study with 140 MFS patients over the age of 18. CT or MRI images were acquired from the base of the skull to the bifurcation of the iliac arteries. They detected aneurysms of the distal aorta and peripheral arteries in 31% of the patients investigated and suggested that, in patients with a prior diagnosis of MFS in cardiological medical follow-up, the main cause of morbidity and mortality is no longer dissection of the ascending aorta, but complications related to aneurysms of the distal and peripheral aorta, showing that it is necessary to search for vascular abnormalities not just in the thoracic aorta, but in the entire body.^15^^,^^17^ Gaertner et al.^10^ used ultrasonography to examine 15 patients with MFS, identifying peripheral aneurysms in 10 subjects (66.7%) and a PAA in 1 of them (6.7%).

## CONCLUSIONS

Over recent decades, reports have emerged of aneurysmal abnormalities associated with MFS. Studies with imaging exams have revealed significant associations with peripheral aneurysms among patients with MFS. The association between the syndrome and PAAs is rare, but its approximate prevalence is difficult to estimate because of the lack of studies with larger numbers of participants. All patients diagnosed with MFS should undergo vascular system investigation as routine.
