# Supplementary material for: Ruptured popliteal artery aneurysm in a patient with a clinical diagnosis of Marfan syndrome
Source: J Vasc Bras. 2020 Oct 16;19:e20200017. doi: 10.1590/1677-5449.200017 (PMC8276642; doi:10.1590/1677-5449.200017)
Supplement: Translation

# Translation: Rotura de aneurisma de artéria poplítea em paciente clinicamente diagnosticado com síndrome de Marfan

**Authors:** Gabriel Paiva Duarte, Jorge Ribeiro da Cunha

**Affiliations:** 1 Universidade Federal Fluminense – UFF, Faculdade de Medicina, Niterói, RJ, Brasil.; 2 Hospital Estadual Alberto Torres – HEAT, São Gonçalo, RJ, Brasil.

**Keywords:** artéria poplítea, aneurisma roto, síndrome de Marfan

## Abstract

A artéria poplítea é o principal local para a ocorrência de aneurismas periféricos. Suas formas de apresentação agudas são potencialmente ameaçadoras à viabilidade do membro e à vida, dentre as quais destacamos a sua rotura. Apesar de ser um evento raro, sua rotura demanda rápida proposta de intervenção para satisfatório desfecho terapêutico. O tratamento padrão-ouro é o cirúrgico convencional e se dá pela interposição de veia safena magna. Trabalhos feitos nas últimas décadas vêm encontrando associações entre a síndrome de Marfan e aneurismas periféricos. Este relato apresenta um caso de um aneurisma de artéria poplítea esquerda roto tratado com sucesso em um paciente de 82 anos diagnosticado clinicamente como portador de síndrome de Marfan previamente desconhecida.

## INTRODUÇÃO

O aneurisma de artéria poplítea (AAP) é o mais comum entre os aneurismas periféricos^1^ ^,^ ^2^ e representa uma ameaça ao membro afetado e, em casos mais graves, à vida do paciente. Os AAPs podem ser assintomáticos^3^ ou se manifestar de forma aguda como emergências médicas, sendo a ocorrência de fenômenos tromboembólicos a causa mais comum e, mais raramente, a sua rotura^2^ ^-^ ^5^.

Sua etiologia é vinculada principalmente à aterosclerose^1^, mas está relacionada também a estados inflamatórios, infecciosos e a doenças do tecido conjuntivo^6^. Há um aumento da prevalência entre homens e idosos^2^, e os principais fatores de risco são hipertensão arterial e tabagismo^4^ ^,^ ^5^. O desenvolvimento dos AAPs na síndrome de Marfan (SM) é raro, existindo poucos casos relatados^7^ ^-^ ^9^. Gaertner et al.^10^ identificaram um paciente com AAP entre 15 com SM submetidos a investigação vascular ultrassonográfica.

## DESCRIÇÃO DO CASO

Um paciente masculino, 82 anos, chegou à emergência com queixa de dor em perna esquerda. Ele relatou surgimento, há 2 anos, de abaulamento doloroso em fossas poplíteas bilateralmente, que aumentou de tamanho desde então. Há cerca de 2 dias, houve aumento significativo do abaulamento em perna esquerda com surgimento de dor. O exame físico mostrou tumor pulsátil em região poplítea esquerda (Figura 1).

**Figura 1:** Paciente no pré-operatório.

O paciente foi submetido à tomografia computadorizada (TC) com contraste, que evidenciou artéria poplítea esquerda (APE) aumentada, com trombo e contraste extravasado, delimitado por um saco (Figura 2), caracterizando-se como um AAP esquerda roto, o qual media 14,4 centímetros em seu maior diâmetro. Após resultado de exames laboratoriais e esclarecimentos prestados, o paciente foi enviado ao centro cirúrgico para imediato reparo do aneurisma roto.

**Figura 2:** Tomografia computadorizada de membro inferior com contraste evidenciando aneurisma de artéria poplítea direita e esquerda, sendo o da esquerda roto.

Optou-se pela abordagem com acesso medial da coxa e perna, com paciente em decúbito dorsal sob anestesia geral. Identificado o saco aneurismático, seguiu-se pela sua dissecção até a identificação das porções proximal e distal da APE. Ambas foram clampeadas para controle de fluxo arterial proximal e distal (Figura 3). O saco aneurismático foi incisado externalizando-se o conteúdo trombótico. Foi feita interposição de prótese de politetrafluoretileno (PTFE) anelado entre os segmentos proximal e distal da APE (Figura 4). O paciente foi submetido previamente à safenectomia, o que impossibilitou o uso de sua veia safena magna (VSM) como enxerto.

**Figura 3:** Saco aneurismático dissecado com controle do fluxo sanguíneo arterial proximal e distal da artéria poplítea esquerda.

**Figura 4:** Anastomose término-terminal de artéria poplítea esquerda com interposição da prótese de politetrafluoretileno (PTFE).

O paciente foi enviado à unidade de terapia intensiva (UTI). Apresentou leucocitose persistente durante internação, e pensamos na possibilidade de infecção da prótese, sendo submetido, no 7º dia, a nova TC com contraste de membros inferiores. O exame mostrou perviedade da prótese, da porção distal da APE e dos seus ramos terminais, a artéria tibial anterior, a artéria tibial posterior e a artéria fibular (Figura 5). A imagem afastou a hipótese de infecção da prótese. O paciente apresentou melhora da leucocitose após antibioticoterapia para tratamento de um quadro de pneumonia e recebeu alta hospitalar no 14º dia após cirurgia.

**Figura 5:** Corte coronal mostrando perviedade da prótese no pós-operatório.

No exame físico inicial, identificamos características físicas da SM, a aracnodactilia e a enoftalmia. Uma semana após a cirurgia, foi realizado novo exame físico, orientado pelos critérios diagnósticos de SM da Ghent Nosology atualizados^11^. O paciente apresentava dilatação da raiz da aorta, com diâmetro de 48 milímetros e um escore-z de raiz de aorta de 4,19. Também apresentava os seguintes achados: sinal de pulso e sinal de punho, deformidade em retropé valgo, assimetria torácica, escoliose toracolombar e protrusão acetabular. Esses achados, em conjunto com um valor de escore-z > 2, cumprem um dos critérios que dão o diagnóstico clínico de SM.

## DISCUSSÃO

A artéria poplítea é o local mais comum de ocorrência de aneurismas periféricos (70 a 85%)^1^ ^,^ ^3^. Nos indivíduos que possuem AAP, há uma alta prevalência de outros aneurismas pelo corpo, como na artéria poplítea contralateral (40 a 68,9%) e na aorta abdominal (30 a 60%)^3^ ^-^ ^5^ ^,^ ^12^ ^,^ ^13^. O desenvolvimento dos AAPs está relacionado à doença aterosclerótica e ocorre em indivíduos com características bem definidas: pacientes do sexo masculino, a partir da 7ª década de vida. Os fatores de risco mais relacionados são a hipertensão arterial e o tabagismo, além de outros menos relacionados, como traumas, estados inflamatórios, infecção por sífilis e doenças do tecido conjuntivo, como a síndrome de Marfan e Ehlers-Danlos^4^ ^,^ ^6^ ^,^ ^12^ ^,^ ^13^.

Clinicamente, os AAPs apresentam-se de forma gradual ou de forma aguda. As apresentações graduais se dão com claudicação intermitente e com sinais compressivos do membro ou até mesmo de forma assintomática. As apresentações ou complicações agudas mais comuns são aquelas relacionadas à interrupção do fluxo arterial, que ocorrem por trombose na artéria ou por embolização distal. Os sintomas são palidez, ausência de pulsos distais e perda súbita de força do membro^1^. A rotura do aneurisma é uma complicação rara dos AAPs (0,5 a 7%)^1^. As apresentações agudas dos AAPs devem ser encaradas como urgências médicas. A pronta resolução se faz necessária pela ameaça à viabilidade do membro e pelo potencial de ameaça à vida do paciente. A confirmação diagnóstica é feita por exames de imagens capazes de detalhar a localização e morfologia da lesão. A TC com contraste, a ultrassonografia duplex-scan, a ressonância magnética (RM) e a angiografia são os exames de escolha^3^ ^,^ ^14^.

O pronto reestabelecimento da perfusão do membro é a base do tratamento de qualquer uma das complicações agudas dos AAPs. Há dois grupos de tratamento propostos: a cirurgia convencional (CC) e os reparos endovasculares (RE)^3^ ^,^ ^14^. A CC é realizada através da anastomose entre os dois segmentos de vaso proximal e distal à lesão ou por interposição de enxerto autólogo ou prótese. O enxerto autólogo mais comumente utilizado é a VSM, enquanto a prótese mais usada é a de PTFE. Em nosso caso, optamos pelo PTFE anelado pelo fato de a anastomose distal ultrapassar a linha articular do joelho; com isso, há um menor risco de dobra do enxerto quando comparado com enxerto não anelado. Os RE baseiam-se na colocação de stents no local da lesão ou na embolização do aneurisma.

O tratamento padrão-ouro é a CC, devendo ser realizada na maioria dos pacientes^3^ ^,^ ^4^. A preferência pelo RE é indicada àqueles pacientes com elevado risco cirúrgico, por ser uma abordagem menos invasiva^6^ ^,^ ^14^. As complicações pós-operatórias mais comuns são as relacionadas à hospitalização (infecções de trato respiratório, insuficiência renal aguda, trombose venosa profunda), seguidas de trombose do enxerto ou prótese, hematomas na região da ferida operatória e problemas de cicatrização^3^. O RE permite que os pacientes sejam submetidos a um menor tempo de internação, com diminuição das complicações da ferida operatória. Já a CC apresenta menor risco de trombose do enxerto venoso ou prótese interposta e melhores resultados na perviedade primária da anastomose a longo prazo^15^.

Na metanálise realizada por Leake et al.^15^, encontrou-se uma taxa de perviedade primária após 3 anos na CC de 79,4%, enquanto no RE essa taxa de perviedade foi de 68,2%. Aulivolla et al.^5^ mostraram que as taxas de perviedade primária após 5 anos foi de 85% na CC e de 44,4% no RE. Restringindo o tratamento ao grupo da CC, Huang et al.^4^ mostraram que a interposição de VSM mostrou superioridade à prótese de PTFE quanto à perviedade primária após 5 anos. A perviedade da VSM foi de 85%, enquanto da prótese de PTFE foi de 50%.

Os AAPs parecem fazer parte de um conjunto de alterações aneurismáticas que afetam outras artérias do corpo. Após correção cirúrgica, 49% dos indivíduos desenvolvem novos aneurismas em um período de 10 anos^2^. Esses pacientes devem ter seu sistema vascular investigado de forma rotineira^2^.

## SÍNDROME DE MARFAN

A SM é uma doença do tecido conjuntivo causada por uma mutação do gene fbn1 e cursa com deformidades ósseas, pulmonares, cardiovasculares e oculares^16^. A mutação no gene fbn1 na SM gera uma alteração da proteína fibrilina, que é um dos componentes da matriz extracelular do tecido conjuntivo das artérias. Atribui-se que as dilatações da parede dos vasos presentes em portadores da síndrome são uma consequência dessa mutação^16^.

Uma das condições associadas à SM é a dilatação de aorta ascendente, enquanto, em outras artérias, há um número restrito de relatos de aneurismas. Foram encontrados relatos de dilatações aneurismáticas em outros segmentos da aorta, em ramos viscerais, artéria pulmonar e artérias periféricas em paciente com SM, mas poucos relatos foram encontrados relacionando a síndrome aos AAPs^7^ ^-^ ^9^ ^,^ ^17^ ^-^ ^19^.

Yetman et al.^17^ realizaram um estudo com 140 pacientes maiores de 18 anos com SM. Foram feitas imagens de TC ou RM da base do crânio à bifurcação das artérias ilíacas. Foram encontrados aneurismas de aorta distal e artérias periféricas em 31% dos pacientes avaliados. Ele sugeriu que, em pacientes com prévio diagnóstico de SM e acompanhamento médico cardiológico, a principal causa de morbidade e mortalidade deixa de ser a dissecção de aorta ascendente e passa a ser complicações relacionadas aos aneurismas de aorta distal e periféricos, levantando a necessidade de pesquisa de alterações vasculares não mais apenas na aorta torácica, mas no corpo todo^15^ ^,^ ^17^. Gaertner et al.^10^ investigaram com ultrassonografia 15 pacientes com SM, tendo identificado aneurismas periféricos em 10 indivíduos (66,7%) e, em 1 deles (6,7%), um AAP.

## CONCLUSÃO

Nas últimas décadas, surgiram relatos de alterações aneurismáticas associadas à SM. Estudos com exames de imagem revelaram significativa associação de aneurismas periféricos entre pacientes portadores de SM. A associação de AAPs na síndrome é rara, porém sua prevalência aproximada é difícil de ser estimada pela carência de estudos com maior número de participantes. Todos os pacientes diagnosticados com SM devem ter seu sistema vascular pesquisado de forma rotineira.
